# Linking Emotional Intelligence, Physical Activity and Aggression among Undergraduates

**DOI:** 10.3390/ijerph182312477

**Published:** 2021-11-26

**Authors:** José Luis Ubago-Jiménez, Mar Cepero-González, Asunción Martínez-Martínez, Fátima Chacón-Borrego

**Affiliations:** 1 Department of Didactics of Musical, Plastic and Corporal Expression, University of Granada, 18071 Granada, Spain; jlubago@ugr.es (J.L.U.-J.); mcepero@ugr.es (M.C.-G.); 2 Department of Research and Diagnosis Methods in Education, University of Granada, 18071 Granada, Spain; asuncionmm@ugr.es; 3 Department of Physical Education and Sports, University of Sevilla, 41013 Sevilla, Spain

**Keywords:** physical activity, emotional intelligence, aggressive behaviours, students

## Abstract

Several indicators are strongly related to health and well-being in university students, such as emotional intelligence and physical activity. At the same time, some qualities threaten it and are incompatible with students’ adaptation to society in general, such as aggressive behaviours. The aim of this research is to analyse the relationship established between emotional intelligence using TMMS-24, physical activity, using IPAQ, and aggression behaviours in university students. For this purpose, a descriptive, cross-sectional and non-experimental study was developed with 932 undergraduates (M = 20.55; SD = 3.673). The findings highlight how emotional intelligence and physical activity practice decrease violent behaviour in university students. In addition, men tend to have more aggressive behaviours than women as well as the relation between physical activity and emotional intelligence is stronger in all its dimensions. Results highlight the importance of including emotional intelligence programs in order to enable undergraduate well-being.

## 1. Introduction

University is a stage of psychophysical transition, where adolescents tend to adopt new habits in order to feel socially included [1]. Likewise, the university period itself generates stressful situations caused by academic performance [2,3].

These stressful situations often induce actions and behaviours that, according to Appelqvist-Schmidlechner et al. [4], are determined by the perception of the individual’s self-concept. The effects of attachment or emotional engagement have also been found in adolescence on their self-confidence, their ability to establish and maintain relationships with others, and their importance and influence in the peer group [5]. Aggressive behaviour in youth is a global public health problem due to its emotional, social, and economic consequences [6].

In this context, physical activity plays a key role in promoting an active and healthy lifestyle in students, with multiple academic, psychosocial, and sport-physical benefits [7,8]. Moderate physical activity generates a multitude of benefits such as reducing cardiovascular diseases [9,10] and gradually lowering levels of obesity and overweight [11]. In addition, in the cognitive sphere, active lifestyles significantly reduce stress and anxiety levels, improving mood and promoting socioaffective and emotional self-regulation, self-esteem, and well-being [12,13,14,15].

These behaviours provoke conflicts like bullying, which is an issue of growing incidence in recent years [16]. This has led to a proliferation in the number of research projects looking for better strategies to address this violence phenomenon. Placing a focus on physical activity, it is found how recent literature reviews [17,18,19] have concluded that through the practice of physical activity, levels of aggression among this population can be significantly reduced.

Sport practice is a way of transmitting values, showing respect for others, as well as compliance with a system or rules [20]. In addition to the mentioned sporting benefits, studies such as Zurita-Ortega et al. [21] have shown how physical activity is a factor in the regulation of aggression among adolescents.

Authors like Singh and Sachdev [22], Vaquero-Solís et al. [23] and Rodríguez-Romo et al. [24] confirm how emotional intelligence (EI) and physical activity are closely related. In fact, the influence exerted by emotional intelligence improves sport performance, which helps to increase levels of satisfaction and decision making among participants in physical activity. On the other hand, although it is known to be important in sports performance [25,26], EI is not seen as a key factor due to insufficient knowledge generated by EI interventions, limited dedication to training, or mistaken thinking about the term [27,28].

External factors such as the place of residence, culture, or society itself can affect all of this. Indeed, some of the aggressive responses of young people are a consequence of the behaviour promoted in the media and social networks.

Taking into account the above, the aim of this research is to analyse the established relationship between EI, physical activity, and aggression in university students. In order to achieve this, the following hypotheses are considered: (a) there is an inverse relationship between EI and aggression; and (b) there is an inverse relationship between physical activity and aggression.

## 2. Materials and Methods

### 2.1. Subjects and Design

Research was conducted following a descriptive, cross-sectional, and non-experimental design with a sample of 932 students from the universities of Almería, Cádiz, Córdoba, Granada, Huelva, Jaén, Málaga, and Sevilla (Spain) using convenience sampling. The departments of corporal expression of different universities were contacted to provide questionnaires to the students. After permission was obtained, students were invited to participate in the research, and 958 students participated, with the inclusion criteria being that the participants had to be signed up in any of the four university courses in the universities described. Its sex-specific distribution is 68.3% (*n* = 637) for women and 31.7% (*n* = 295) for men. 

More specifically, the average age of the participants was between 18 and 28 years old (20.55 ± 3.673). It is also to be noted that 26 questionnaires were invalidated due to some errors in their completion, missing items, or duplicate answers.

### 2.2. Instruments

Four instruments were used in this investigation. An ad hoc questionnaire was used to collect sociodemographic data (sex and age).

The second one is the International Physical Activity Questionnaire (IPAQ), which was developed in Geneva in 1998 [29] and it is one of the most widely used questionnaires to measure physical activity levels in a specific sample. This questionnaire is divided into four domains (work, transport, home activities, and leisure time), where the frequency and duration of physical activity lasts for more than 10 min. The final result is obtained in three categories: vigorous (VPA), moderate (MPA), and light (LPA) activities. For the present study, the results obtained a reliability of α = 0.891.

The Trait Meta-Mood Scale-24 (TMMS-24), by Fernández-Berrocal et al. [30], was used to measure EI. Its original version is the one developed by Salovey et al. [31]. This tool has been used in many social science research contexts. The reliability obtained in our study is of α = 0.901 for the dimension ‘Emotional Attention’ (EA); α = 0.887 and α = 0.915 for ‘Clarity feelings’ (CF); and for ‘Emotional Repair’ (ER) some indexes of α = 0.871.

For the measurement of violent behaviour, the “Violent Behavior at School Scale”, proposed in its original version by Little et al. [32] and adapted to Spanish by Estévez [33] was used. This questionnaire is divided into two categories: manifest aggression and relational aggression, which are further subdivided into three subscales: pure, reactive, and instrumental. It is also answered by means of a 25-item Likert-type scale ranging from 1 = never to 4 = always. Once the scale is completed, two types of violent behaviour are obtained: manifest aggression, which is generated in a personal encounter between the aggressor and the victim; and relational aggression, which is considered when the aggressor remains anonymous. Reliability in the present study was obtained with a Cronbach’s Alpha of α = 0.798 for items measuring manifest aggression and α = 0.735 for relational aggression.

### 2.3. Procedure

In order to conduct the study, the procedure was carried out in several stages. The first step was to request a research authorisation and ethics committee from the Faculty of Education Sciences of the University of Granada (Granada, Spain), obtaining the permission code 1478/CEIH/2020. The second phase was to draft a research document explaining scientific aims and research topics, and to request consent for the students’ participation. After they agreed to participate in the study, the questionnaire was sent by email for honest completion. 

The study was completed by 958 undergraduate students, and 26 questionnaires had to be eliminated because they were not properly completed. Data collection was carried out between May and June 2021, ensuring that the confidentiality of the answers was guaranteed. Data collection and analysis were carried out in accordance with the ethical principles established by the Declaration of Helsinki in 1975 and its update carried out in Brazil in 2013.

### 2.4. Statistical Analysis

IBM SPSS 25.0 statistical software (International Business Machines Corporation, Armonk, NY, USA) was used during the data analysis process. The means and frequencies of the variables were calculated. To obtain the effect size, Cohen’s standardised d was used, which can be interpreted as zero effect (0–0.19); low effect (0.20–0.49); medium effect (0.50–0.79); or high effect (≥0.80). The 95% confidence interval was also determined.

For testing and understanding differences between correlations, the effect size was calculated with Cohen’s q. In addition, Fisher’s Z was calculated by subtracting both correlations from each other. Furthermore, to understand this measurement, the parameters: no effect (≤0.1); small effect (0.1–0.3); medium effect (0.3–0.5); and large effect (0.5) were established. Differences between categorical variables were calculated using contingency tables. On the other hand, ANOVA and Student’s *t*-test were used to analyse differences between categorical and interval variables. In addition, Bonferroni’s test was used to check for intergroup differences. Finally, the linear regression analysis was designed to establish and understand the association between EI and physical activity (independent variables) and aggressive behaviours (dependent variable), adjusted for sex.

## 3. Results

Table 1 shows the results achieved in the variables of the present investigation in relation to the sex of the participants. Statistically significant relationships were found in MPA and VPA (*p* = 0.001; *p* = 0.013) for men. For EI, men showed higher average values for ER (M = 3.72; SD = ±0.721) (*p* = 0.004) and CF (M = 3.66; SD = ±0.729) (*p* = 0.007) than women. There are also higher mean values for women in EA, (M = 4.08; SD = 0.712) (*p* = 0.001).

In relation to aggressive behaviours, statistically significant relationships are found in all dimensions with higher values for men. MA values (M = 1.30; SD = 0.330 vs. M = 1.24; SD = 0.270) representing a small effect size (d = 0.207); PMA (M = 1.54; SD = 0.490 vs. M = 1.43; SD = 0.430) representing a small effect size (d = 0.245); RMA (M = 1.08; SD = 0.209 vs. M = 1.05; SD = 0.177) representing a small effect size (d = 0.160); IMA (M = 1.31; SD = 0.353 vs. M = 1.26; SD = 0.312) representing a small effect size (d = 0.154); RA (M = 1.51; SD = 0.449 vs. M = 1.43; SD = 0.405) representing a small effect size (d = 0.191); PRA (M = 1.16; SD = 0.315 vs. M = 1.09; SD = 0.247) representing a small effect size (d = 0.259); RRA (M = 1.29; SD = 0.259 vs. M = 1.23; SD = 0.220) representing a small effect size (d = 0.257); and IRA (M = 1.33; SD = 0.304 vs. M = 1.26; SD = 0.267) representing a small effect size (d = 0.251). 

The correlational analysis between the research variables for women is shown in Table 2. The strongest relationships are found between the dimensions of VPA and EI, CF (r = 0.722), EA (r = 0.704), and GEI (r = 0.686). Likewise, in terms of MPA, a moderate relationship is found with GEI (r = 0.612) and EA (r = 0.478). On the other hand, a strong relationship is found between LPA and GEI (r = 0.627).

The correlational analysis between the research variables for men is shown in Table 3. Negative and direct relationships were obtained for physical activity and aggressive behaviours. For VPA practice, a negative relationship was found with RMA (r = −0.134), IMA (r = −0.118), PMA (−0.111), MA (r = −0.105), and RA (r = −0.058). For MPA practice with MA (r = −0.097), RMA (r = −0.072), PMA (r = −0.057), RA (r = −0.041), PRA (r = −0.033), and IMA (r = −0.029). Additionally, negative relationships were also found between LPA and MA (r = −0.068), IMA (r = −0.054), RMA (r = −0.041), PMA (r = −0.033) and RA (r = −0.015).

The correlations between EI and aggressive behaviours also reported negative and direct relationships between GEI and IMA (r = −0.126), MA (r = −0.118), and RMA (r = −0.099). The dimension of ER was related to IMA (r = −0.154), MA (r = −0.137), and PMA (r = −0.130). For the CF dimension, a negative relationship was found with RRA (r = −0.118).

The correlations for men reported slightly higher levels than for women. The relationships between the VPA and EI dimensions reported strong correlations with CF (r = 0.489), EA (r = 0.766), GEI (r = 0.712), and ER (r = 0.521). Similarly, MPA reported correlations with GEI (r = 0.697), EA (r = 0.511), ER (r = 0.389), and CF (r = 0.324); while LPA correlated more strongly with GEI (r = 0.658) and ER (r = 0.350).

In relation to physical activity and aggressive behaviours, negative and direct relationships were obtained. VPA was related to MA (r = −0.176), RMA (r = −0.136), PMA (r = −0.124), IMA (r = −0.105), and RA (r = −0.092). MPA was correlated with MA (r = −0.115), RMA (r = −0.098), PMA (r = −0.065), IMA (r = −0.078), RA (r = −0.063), and PRA (r = −0.028). LPA correlated with MA (r = −0.124), IMA (r = −0.089), RMA (r = −0.047), and PMA (r = −0.025).

Finally, the relationships between EI and aggressive behaviours were negatively presented. GEI was correlated with MA (r = −0.112), PMA (r = −0.123), RMA (r = −0.071), and IMA (r = −0.073). RE was correlated with PMA (r = −0.154), MA (r = −0.127), IMA (r = −0.113), PRA (r = −0.086), RA (r = −0.080) and RRA (r = −0.066). Finally, CF was correlated with PMA (r = −0.097) and MA (r = −0.078).

Linear regression analyses were performed to verify the association between EI, physical activity, and aggressive behaviours. Regression was also carried out to differentiate between men and women (Table 4). For predictive models in relation to men, EI and physical activity are a predictive variable of MA (β = 0.177; *p* = 0.015; β = 0.184; *p* = 0.018), which explains 32% of the variance of the response variables; for the PMA dimension (β = 0.155; *p* = 0.009; β = 0.188; *p* = 0.016) explaining 20% of the variance; RMA (β = 0.028; *p* = 0.008; β = 0.034; *p* = 0.020) predictive value decreases to 15%; and EI is a predictor (2%) of the IMA dimension (β = 0.151; *p* = 0.024).

On the other hand, in the case of women, physical activity was a predictive variable of PMA (β = 0.263; *p* = 0.025), explaining 17% of the variance, and EI was a predictive variable of RMA (β = 0.375; *p* = 0.002) explaining 15% of the variance. For the remaining dimensions of aggressive behaviours, none of the introduced variables were significant predictors. 

## 4. Discussion

According to the purpose of the study, the relationship between EI, physical activity, and aggressive behaviours in university students has been analysed; highlighting how EI and the practice of physical activity decrease violent behaviour in university students.

As a general observation, based on the results obtained, it has been observed that men have higher values in all dimensions of aggressive behaviour than women. Likewise, men have higher aggressive levels than women in both their manifest and relational expressions. Similar results are consistent with most research studies on sex differences related to aggressive behaviour [21,34,35,36,37,38,39,40]. In contrast, these findings are opposed to those reflected in the study by Blasco and Orgilés [41], who found that women were more inclined to physical aggression than men in a population of football players between 7 and 17 years old.

Considering relationships between EI and aggressive behaviours, weak negative relationships were found according to previous studies [42]. Students with high levels of GEI have lower rates of manifest aggression and its dimensions. It can also be observed that students with higher scores on the ER dimension have lower scores for both manifest and relational aggression. These data are consistent with studies such as Bibi et al. [43] and Antoñanzas [44], which found inverse relationships between EI and aggressive behaviours. Alvarado et al. [45] and Segura et al. [46] have shown that young people with a higher level of EI are less likely to show any type of aggression.

The relationship between physical activity practice and aggressive behaviours shows how physical activity practice in its three modalities (VPA, MPA, and LPA) reduces aggressive behaviours. Likewise, it shows that students’ aggressive behaviour decreases with higher levels of physical activity. The findings show that adherence to physical activity helps reduce aggressive behaviour [47,48,49]. Taking into account that physical activity practice is a factor to reduce stress and release tension, it could also be said to reduce aggressive behaviour. Furthermore, studies such as Park et al. [50] and Jenkins et al. [51] show how physical activity reduces aggression among adolescents.

In addition, the relationship between EI and PA was found to be direct and strong for the sample surveyed. In particular, it revealed that students who practiced more physical activity had higher levels of general emotional intelligence, as well as all its dimensions. These results are consistent with previous studies such as those conducted by Wang et al. [28] and Acebes et al. [52] or the one developed by Roy et al. [53].

Moreover, it is necessary to point out the limitations associated with this research. First of all, it was conducted with undergraduates in order to match a sample with similar research projects. Furthermore, it was a descriptive and cross-sectional design and convenience sampling which gives valuable information concerning current issues, but also avoids casual conclusions. Another limitation concerns the instruments used, since, although they have a high reliability, it would have been interesting to measure physical activity with accelerometry instead of a reported test. Recognising the above limitations, the following suggestions are made for future research. The research should be replicated in and extended to other countries with similar socio-economic characteristics. In addition, it would be interesting to develop an intervention program to test a combined influence of EI and PA on the reduction of aggressive behaviour in university students.

## 5. Conclusions

Principal findings showed how physical activity and EI help reduce aggressive behaviours. Additionally, IE was negatively associated with aggressive behaviours, suggesting a greater ability to control emotions, which means lower problems associated with aggressive behaviours. These negative relationships found between EI point to an important point to address. According to initial hypotheses, it was found that the higher the physical activity intensity, the lower the aggressiveness indexes. It is necessary to emphasize physical activity not only to improve or maintain physical health, but also to improve one’s own behaviour, since it releases accumulated stress. In fact, the main implications of the present study are oriented to the consolidation of evidence for designing and developing psychophysical-healthy programs in higher education.

## Figures and Tables

**Table 1:** EI, aggressive behaviours and PA practice according to participants’ sex.

Variable	Men		Women		Levene’s Test		Sig.	Es (d)	95% CI
	M	S.D.	M	S.D.	F	Sig.	(Bilateral)		
LPA	754.2	1198	542.8	994.3	5.238	0.075	0.854	0.199	(0.061; 0.337)
MPA	1045.6	1587.2	631.5	1240.8	6.324	0.001	0.001 *	0.305	(0.166; 0.443)
VPA	264.5	793.1	82.3	421.7	2.549	0.104	0.013 *	0.322	(0.183; 0.461)
GEI	3.72	0.537	3.70	0.575	1.625	0.203	0.477	0.036	(−0.103; 0.174)
ER	3.72	0.721	3.57	0.811	5.110	0.024	0.004 *	0.202	(0.063; 0.34)
CF	3.66	0.729	3.52	0.814	4.468	0.035	0.007 *	0.178	(0.039; 0.316)
EA	3.78	0.745	4.08	0.712	0.649	0.421	0.001 *	−0.415	(−0.554; −0.276)
MA	1.30	0.330	1.24	0.270	22.013	0.001	0.009 *	0.207	(0.068; 0.345)
PMA	1.54	0.490	1.43	0.430	13.683	0.001	0.001 *	0.245	(0.106; 0.383)
RMA	1.08	0.209	1.05	0.177	18.325	0.001	0.016 *	0.160	(0.022; 0.298)
IMA	1.31	0.353	1.26	0.312	10.251	0.001	0.035 *	0.154	(0.015; 0.292)
RA	1.51	0.449	1.43	0.405	1.951	0.163	0.010 *	0.191	(0.052; 0.329)
PRA	1.16	0.315	1.09	0.247	25.350	0.001	0.001 *	0.259	(0.120; 0.397)
RRA	1.29	0.259	1.23	0.220	21.296	0.001	0.001 *	0.257	(0.119; 0.396)
IRA	1.33	0.304	1.26	0.267	9.592	0.002	0.001 *	0.251	(0.112; 0.389)

Note: low physical activity (LPA); moderate physical activity (MPA); vigorous physical activity (VPA); general emotional intelligence (GEI); emotional repair (ER); clarity feelings (CF); emotional attention (EA); manifest aggression (MA); pure manifest aggression (PMA); reactive manifest aggression (RMA); instrumental manifest aggression (IMA); relational aggression (RA); pure relational aggression (PRA); reactive relational aggression (RRA); instrumental relational aggression (IRA) * *p* < 0.05.

**Table 2:** Bivariate correlations between PA, EI, and aggressive behaviours for women.

	LPA	MPA	VPA	GEI	ER	CF	EA	MA	PMA	RMA	IMA	RA	PRA	RRA	IRA
**LPA**	-	0.394 **	0.455 **	0.627 **	0.338 **	0.210 **	0.124 **	−0.068 **	−0.033 **	−0.041 **	−0.054 **	−0.015 **	−0.014	−0.018	−0.057
**MPA**		-	0.502 **	0.612 **	0.299 **	0.358 **	0.478 **	−0.097 **	−0.057 **	−0.072 **	−0.029 **	−0.041 **	−0.033 **	−0.019	−0.015
**VPA**			-	0.686 **	0.493 **	0.722 **	0.704 **	−0.105 **	−0.111 **	−0.134 **	−0.118 **	−0.058 **	−0.022	−0.004	−0.050
**GEI**				-	0.697 **	0.823 **	0.682 **	−0.118 **	−0.055	−0.099 *	−0.126 *	−0.070	−0.048	−0.094	−0.015
**ER**					-	0.438 **	0.109	−0.137 **	−0.130 **	−0.066	−0.154 **	−0.081	−0.112	−0.090	0.018
**CF**						-	0.378 **	−0.086	−0.046	−0.060	−0.108	−0.082	−0.035	−0.118 *	−0.030
**EA**							-	−0.038	−0.052	−0.092	−0.017	0.007	0.039	−0.002	−0.020
**MA**								-	0.767 **	0.830 **	0.695 **	0.713 **	0.579 **	0.612 **	0.546 **
**PMA**									-	0.376 **	0.502 **	0.547 **	0.496 **	0.408 **	0.445 **
**RMA**										-	0.353 **	0.527 **	0.361 **	0.554 **	0.341 **
**IMA**											-	0.607 **	0.478 **	0.450 **	0.611 **
**RA**												-	0.819 **	0.878 **	0.757 **
**PRA**													-	0.558 **	0.474 **
**RRA**														-	0.502 **
**IRA**															-

Note: low physical activity (LPA); moderate physical activity (MPA); vigorous physical activity (VPA); general emotional intelligence (GEI); emotional repair (ER); clarity feelings (CF); emotional attention (EA); manifest aggression (MA); pure manifest aggression (PMA); reactive manifest aggression (RMA); instrumental manifest aggression (IMA); relational aggression (RA); pure relational aggression (PRA); reactive relational aggression (RRA); instrumental relational aggression (IRA) **: correlation significant at the 0.01 level (bilateral); *: correlation significant at the 0.05 level (bilateral).

**Table 3:** Bivariate correlations between PA, EI, and aggressive behaviours for men.

	LPA	MPA	VPA	GEI	ER	CF	EA	MA	PMA	RMA	IMA	RA	PRA	RRA	IRA
**LPA**	-	0.462 **	0.345 **	0.658 **	0.350 **	0.125 **	0.103 **	−0.124 **	−0.025 **	−0.047 **	−0.089 **	−0.005	−0.007	−0.005	−0.102
**MPA**		-	0.568 **	0.697 **	0.389 **	0.324 **	0.511 **	−0.115 **	−0.065 **	−0.098 **	−0.078 **	−0.063 **	−0.028 **	−0.009	−0.022
**VPA**			-	0.712 **	0.521 **	0.789 **	0.766 **	−0.176 **	−0.124 **	−0.136 **	−0.105 **	−0.092 **	−0.031	−0.012	−0.037
**GEI**				-	0.735 **	0.821 **	0.634 **	−0.112 **	−0.123 **	−0.071 *	−0.073 *	−0.050	−0.046	−0.044	−0.032
**ER**					-	0.466 **	0.119 **	−0.127 **	−0.154 **	−0.056	−0.113 *	−0.080 *	−0.086 **	−0.066 *	−0.044
**CF**						-	0.315 **	−0.078 *	−0.097 **	−0.053	−0.031	−0.031	−0.022	−0.045	−0.002
**EA**							-	−0.037	−0.014	−0.047	-0.014	0.004	0.010	0.016	-0.025
**MA**								-	0.746 **	0.839 **	0.704 **	0.705 **	0.545 **	0.634 **	0.547 **
**PMA**									-	0.360 **	0.487 **	0.536 **	0.480 **	0.438 **	0.408 **
**RMA**										-	0.353 **	0.527 **	0.361 **	0.554 **	0.341 **
**IMA**											-	0.607 **	0.478 **	0.450 **	0.611 **
**RA**												-	0.819 **	0.878 **	0.757 **
**PRA**													-	0.558 **	0.474 **
**RRA**														-	0.502 **
**IRA**															-

Note: low physical activity (LPA); moderate physical activity (MPA); vigorous physical activity (VPA); general emotional intelligence (GEI); emotional repair (ER); clarity feelings (CF); emotional attention (EA); manifest aggression (MA); pure manifest aggression (PMA); reactive manifest aggression (RMA); instrumental manifest aggression (IMA); relational aggression (RA); pure relational aggression (PRA); reactive relational aggression (RRA); instrumental relational aggression (IRA) **: correlation significant at the 0.01 level (bilateral); *: correlation significant at the 0.05 level (bilateral).

**Table 4:** Regression model for EI, PA and aggressive behaviours.

Variables	Standardized *β*		*t*		*p*		95% CI		R^2^		Adjusted R^2^	
	Men	Women	Men	Women	Men	Women	Men	Women	Men	Women	Men	Women
Manifest Aggression									0.244	0.147	0.327	0.107
EI	0.177	0.125	−1.127	−1.096	0.015	0.078	(−4.05; 0.46)	(−3.87; 0.35)				
PA	0.184	0.322	−0.251	−0.285	0.018	0.095	(−1.74; 1.68)	(−1.55; 1.22)				
Pure Manifest Aggression									0.256	0.122	0.207	0.174
EI	0.155	0.215	−0.742	−0.718	0.009	0.257	(−3.83; 0.21)	(−3.64; 0.31)				
PA	0.188	0.263	0.459	0.426	0.016	0.025	(−1.08; 1.98)	(−1.14; 1.33)				
Reactive Manifest Aggression									0.127	0.102	0.152	0.148
EI	0.028	0.375	0.108	0.106	0.008	0.002	(−1.51; 1.58)	(−1.28; 1.42)				
PA	0.034	0.204	0.164	0.091	0.020	0.075	(−1.97; 0.73)	(−1.84; 0.85)				
Instrumental Manifest Aggression									0.192	0.130	0.156	0.025
EI	0.181	0.284	−0.401	−0.619	0.024	0.119	(−1.67; 0.59)	(−1.74; 0.62)				
PA	0.158	0.312	−0.318	−0.521	0.062	0.317	(−1.87; 1.52)	(−1.48; 1.05)				
Relational Aggression									0.108	0.161	0.198	0.119
EI	0.479	0.252	0.715	0.721	0.651	0.322	(−1.09; 3.11)	(−1.12; 2.54)				
PA	0.577	0.547	0.657	0.643	0.346	0.218	(−1.23; 1.97)	(−1.08; 1.67)				
Pure Relational Aggression									0.141	0.129	0.123	0.097
EI	0.804	0.486	0.741	0.628	0.163	0.412	(−2.87; 1.54)	(−1.91; 1.23)				
PA	0.454	0.198	0.903	0.352	0.198	0.235	(−2.46; 0.89)	(−2.32; 1.43)				
Reactive Relational Aggression									0.133	0.196	0.088	0.103
EI	0.642	0.162	0.195	0.224	0.781	0.741	(−1.82; 2.20)	(−1.84; 2.01)				
PA	0.511	0.273	1.751	0.714	0.296	0.263	(−0.55; 2.51)	(−0.84; 1.88)				
Instrumental Relational Aggression									0.247	0.267	0.065	0.010
EI	0.386	0.278	0.133	−0.242	0.683	0.801	(−1.78; 2.12)	(−1.66; 2.03)				
PA	0.891	0.871	0.264	−0.103	0.874	0.172	(−1.33; 1.63)	(−1.42; 1.52)				

Note: *p* < 0.05; physical activity (PA); emotional intelligence (EI).
